# A Rare Case of Superior Vena Cava Syndrome in a Patient With Rheumatoid Arthritis and IgA Nephropathy

**DOI:** 10.7759/cureus.28198

**Published:** 2022-08-20

**Authors:** Colton M Moore, Autumn Loichle, Kameron Tavakolian, Mihir Odak, Savannah Nightingale, Swapnil V Patel

**Affiliations:** 1 Internal Medicine, St. George's University School of Medicine, Neptune, USA; 2 Internal Medicine, Jersey Shore University Medical Center, Neptune City, USA; 3 Department of Obstetrics and Gynecology, Monmouth Medical Center, Long Branch, USA; 4 Internal Medicine, Jersey Shore University Medical Center, Neptune, USA

**Keywords:** thrombosis, iga nephropathy, rheumatoid vasculitis, rheumatoid arthritis, superior vena cava (svc) syndrome

## Abstract

Superior vena cava syndrome (SVCS) is a vascular condition resulting from an impaired venous return to the right atrium. The majority of SVCS cases are caused by mass effect in which extrinsic compression of the vessel leads to obstruction of blood flow. In less common cases of SVCS, thrombus formation and luminal narrowing can result in poor return through the SVC. Inflammatory causes of SVCS are even rarer and poorly documented. IgA nephropathy and rheumatoid arthritis (RA) are two autoimmune diseases with the potential to cause vasculitis, thus increasing the likelihood of intraluminal vessel occlusion. We report a rare case of SVCS in a 65-year-old female with a past medical history significant for atrial fibrillation, IgA nephropathy, chronic kidney disease stage IIIA, and RA who presented with headache, dizziness, and neck pain and swelling extending down the left upper extremity for three days. Inflammatory SVCS is uncommon and cases of SVCS secondary to RA and IgA nephropathy are underreported in the literature thus far. Our hope in presenting this case is to encourage a greater degree of suspicion for vascular complications, such as SVCS, in patients with autoimmune and inflammatory conditions.

## Introduction

Superior vena cava syndrome (SVCS) is a condition that results from impaired venous return through the superior vena cava (SVC) to the right atrium. More than 80% of the etiologies of SVCS result from malignancy [1]. On the contrary, intraluminal etiologies account for a minority of cases and can be from inflammatory luminal narrowing or thrombus formation [2]. SVCS patients usually present with facial, neck, and upper extremity swelling, severe forms of SVCS can progress to cerebral edema and upper respiratory compromise [3]. Imaging modalities that aid in diagnosis includes contrast-enhanced computed tomography (CT), magnetic resonance imaging, venography, and ultrasonography with Doppler. Ultimately in all cases of SVCS, treatment is accomplished by relieving the underlying obstruction, (e.g., tumor-specific management, anticoagulation of thrombosis, balloon angioplasty, stent placement) improving flow through the SVC [4]. 

Although there are numerous autoimmune diseases that cause inflammation of vessel walls, literature documenting inflammatory SVCS is limited. Rheumatoid vasculitis (RV) is a vascular sequela of RA, where inflammation leads to necrosis of small and medium-sized blood vessels [5]. IgA nephropathy involves the circulation of antibodies and increases a patient’s inflammatory state, with the potential to cause autoantibody-mediated endothelial damage to vessel walls [6]. Here, we report a rare case of inflammatory SVCS highly suspected to be secondary to RA and IgA nephropathy.

## Case presentation

A 65-year-old female with a medical history significant for atrial fibrillation on rivaroxaban, rheumatoid arthritis, IgA nephropathy, chronic kidney disease (CKD) stage IIIA, chronic obstructive pulmonary disease, type II diabetes mellitus, and morbid obesity presented with progressive headache, dizziness, neck pain, and swelling extending down to the left upper limb for three days. Her dizziness was described as if the room was spinning and was worsened by standing and improved with rest. She also endorsed tinnitus, however, reported no changes in vision, chest pain, dysphagia, fever, chills, dyspnea, or swelling of any other extremities. The patient did not use oral contraceptives or hormonal replacement therapy. Additionally, she denied any history of pregnancy complications or a family history of thrombophilic disorders. 

**Figure 1:**
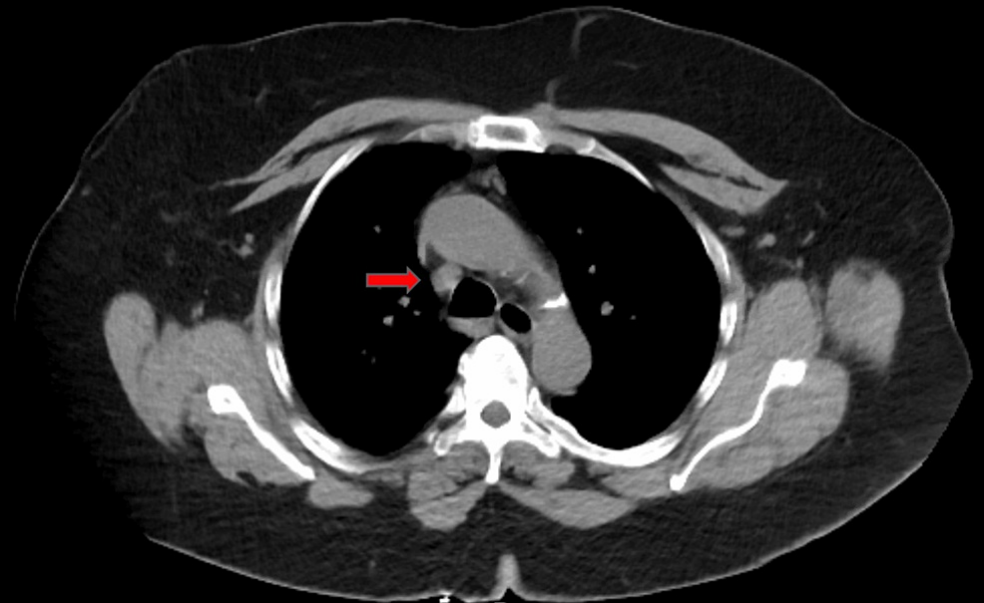
CT scan of the chest without contrast showing narrowing of the superior vena cava at the level of the aortic arch (red arrow).

**Figure 2:**
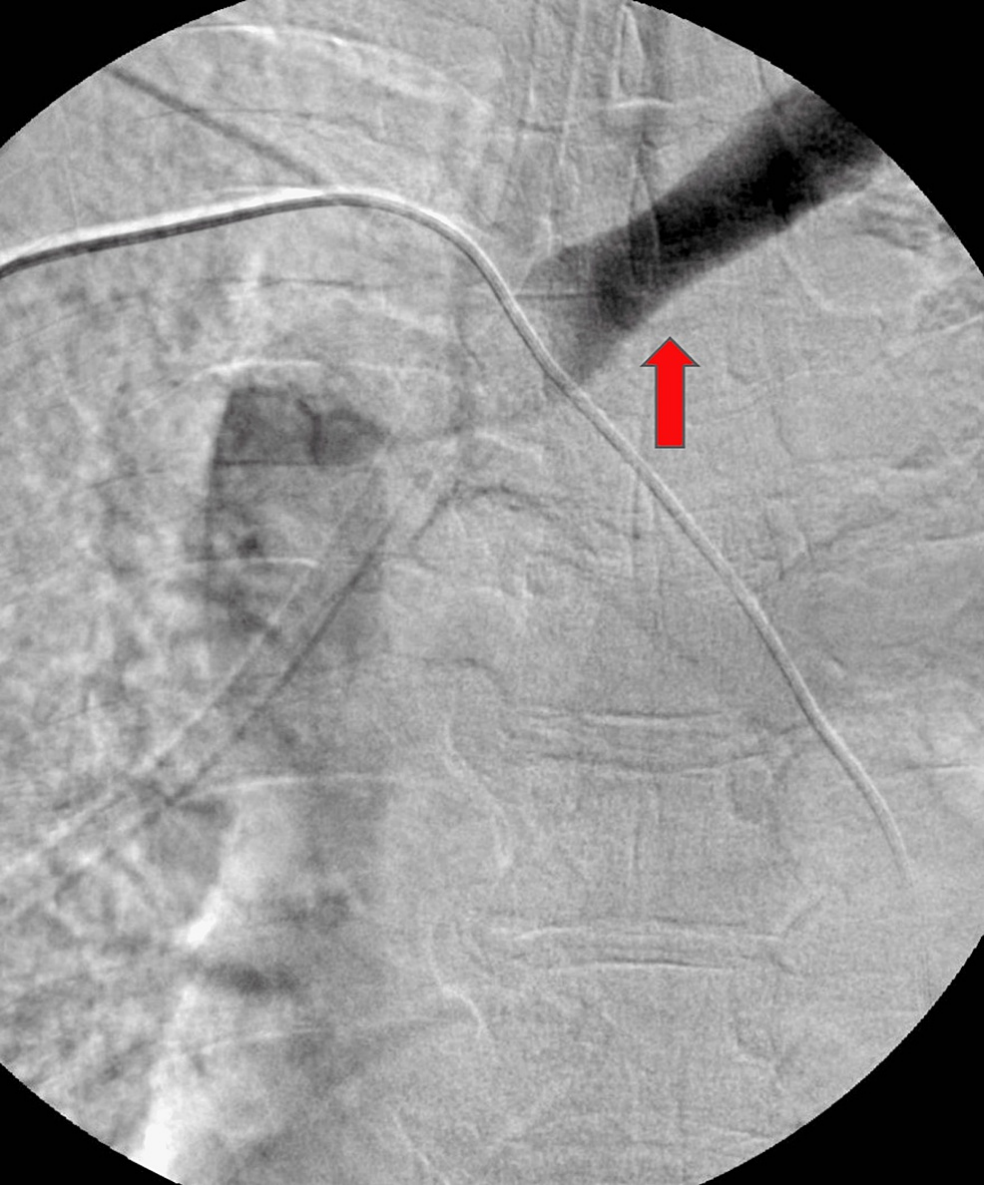
Venogram showing circumferential narrowing of the proximal left innominate vein (red arrow).

Upon presentation, her vital signs were all within normal limits. Her examination was significant for swelling and tenderness of the anterior and posterior neck with a limited range of motion secondary to pain. The patient also had diffuse swelling and tenderness of the left upper extremity extending from the shoulder to the fingers. Her initial laboratory studies (Table 1) were notable for elevated creatinine and D-dimer. A contrast-free CT scan of the chest (Figure 1) was performed, which revealed marked narrowing of the SVC at the level of the aortic arch without any compressive mass. Vascular surgery was consulted and the decision was made to perform a venogram (Figure 2), which revealed a patent SVC and near occluded left innominate vein in a symmetric manner that appeared to be inflammatory in nature. Balloon angioplasty was performed and repeated venogram showed improvement in flow. The following day, the patient experienced an improvement in her symptoms and she was discharged with outpatient follow-up.

**Table 1:** Initial laboratory results

Serum	Results	Reference range
White Blood Cells (x10^3^/uL)	7.2	4.5 - 11.0
Hemoglobin (g/dL)	11.6	12.0 - 16.0
Mean Corpuscular Volume (fL)	88.4	80.0 - 100.0
Platelet Count (x10^3^/uL)	165	140 - 450
Glucose (mg/dL)	209	136 - 145
Blood Urea Nitrogen (mg/dL)	20	5 - 25
Creatinine (mg/dL)	1.46	0.44 - 1.0
Sodium (mmol/L)	136	135 - 146
Potassium (mmol/L)	5.2	3.5 - 5.2
Chloride (mmol/L)	105	96 - 110
Calcium (mg/dL)	9.2	8.5 - 10.5
Magnesium (mg/dL)	2.0	1.3 - 2.5
Bicarbonate (mmol/L)	25	24 - 31
Alkaline phosphatase (U/L)	74	38 - 126
Total protein (g/L)	6.6	6.0 - 8.0
Albumin (g/dL)	3.7	3.5 - 5
Bilirubin, Total (mg/dL)	.9	0.2 - 1.3
Aspartate aminotransferase (U/L)	32	10 - 42
Alanine aminotransferase (U/L)	20	10 - 60
Troponin (ng/mL)	0.02	<0.04

**Figure 3:**
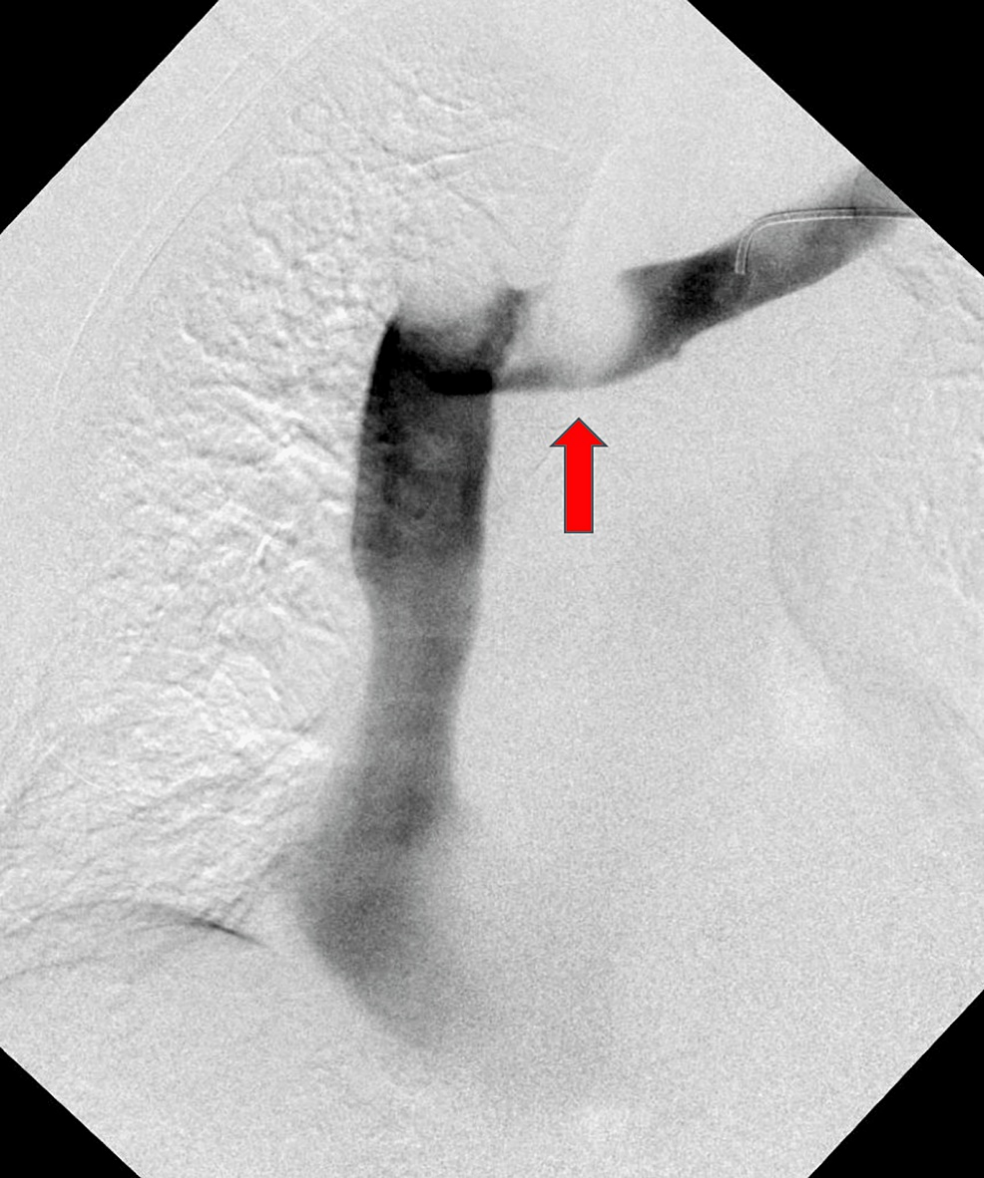
Venogram showing a thrombus in the proximal left innominate vein (red arrow).

Two weeks later, the patient returned with complaints of worsening dizziness on exertion. Associated symptoms included blurry vision, headache, neck pain, and swelling similar to her initial presentation. Physical examination revealed neck, facial, and left upper extremity swelling. A venogram (Figure 3) was performed and revealed what appeared to be a large thrombus of the left innominate vein. The decision was made to perform a thrombectomy and balloon angioplasty. After the complete resolution of her symptoms, she was discharged on apixaban 5 milligrams twice daily and was advised to follow up outpatient with rheumatology, hematology, and vascular surgery.

## Discussion

SVCS results from an impedance of blood flow through the SVC to the right atrium. The majority of cases result from extraluminal compression via mass effect. Rarely, inflammatory autoimmune conditions affecting blood vessel walls can lead to decreased lumen diameter and SVCS. RV is an extra-articular manifestation of RA that causes inflammation and narrowing of small and medium-sized vessels via immune complex deposition in vessel walls [7]. Although most cases of RV cause small-vessel stenosis via reactive intimal tissue, cases of mesenteric vasculitis as well as hepatic arteritis have been reported [8]. A case published in 2021 documented a patient with RV and antiphospholipid antibody syndrome presenting with inflammatory stenosis (confirmed with artery biopsy) of the femoral artery and widespread vasculitis of the deep muscular arteries and veins [9]. There have been only a handful of cases of SVCS in patients with RA. All but one of the cases involved thrombotic pathogenesis secondary to diseases such as antiphospholipid antibody syndrome and systemic lupus erythematosus [10]. The absence of a secondary “pro-thrombic” disease emphasizes the rarity of our case in that it is an inflammatory rather than thrombotic pathogenesis of SVCS.

Similar to RV, IgA nephropathy damages the vasculature in the kidneys by depositing immune complexes composed of IgA. The buildup of these immune complexes activates the complement system and aggravates neutrophils which further mount an inflammatory response in the vessel wall [11]. Although IgA nephropathy mainly impacts the vasculature of the kidneys, severe disease can lead to high levels of circulating oxidized free radicals and inflammatory cytokines such as interleukin-1, interleukin-6, and tumor necrosis factor-alpha, which can cause systemic inflammation [12]. There are no reported cases of inflammatory-mediated SVCS in patients with IgA nephropathy, however, there are reports of thrombus formation in patients with nephrotic syndromes that lead to SVCS [13]. Previous literature is more suggestive of hypercoagulable pathogenesis to vessel occlusion rather than inflammatory stenosis in patients with IgA nephropathy [6]. 

Management of SVCS first starts with identifying the intravascular or extravascular cause of obstruction via imaging, commonly chest x-ray or CT angiography. In unstable patients, such as those with cerebral or laryngeal edema, securing the airway, breathing, and circulation takes precedence. Once stable, angioplasty with or without stent placement to achieve endovascular recanalization is the mainstay of treatment [4]. If SVC obstruction is due to thrombosis, catheter-directed thrombolysis with or without stent placement may be preferred [4]. Traditionally, malignant causes of SVCS were managed with radiation therapy and surgical removal of masses, however, the endovascular intervention has proven to provide faster relief and clinical improvement in patients [4]. Although both balloon dilation and stenting improve blood flow through the vessel lumen; studies comparing balloon dilation versus stent placement found that patients who underwent balloon dilation required significantly more re-interventions compared to patients with stent placement alone [14]. Most re-stenosis of the vessel after balloon dilation requiring second interventions occurred within the first week of primary intervention [14]. The argument can be made that our patient may have benefitted from primary intervention with balloon angioplasty and stent placement, decreasing the likelihood of her requiring re-intervention. 

Our patient’s past medical history of RA elevated her risk for thrombosis even on sufficient anticoagulation. Many studies have established the procoagulant state in patients with RA. A study by Beata Kwasny-Krochin, et al. concluded that patients with RA have altered fibrin clot properties resulting in an increased likelihood of thrombus formation as compared to patients without RA with no other cardiovascular risk factors [15]. Another study investigated the role of increased expression of extracellular vesicles in the plasma of patients with RA. This study confirmed the majority of these vesicles were platelet in origin and have phospholipid membranes that act as a catalyst for the activation of the coagulation processes leading to increased thrombin and fibrin formation [16]. In our patient, thrombus formation within the brachiocephalic vein was likely related to the recent balloon angioplasty in the presence of a hypercoagulable state secondary to RA and IgA nephropathy.

## Conclusions

Patients with SVCS rarely present with intravascular causes of obstruction. It is important to conduct CT angiograms or venograms in patients with high suspicion for SVCS who have pro-inflammatory conditions (e.g. RA and IgA nephropathy) as these patients can have circumferential vessel wall inflammation leading to decreased perfusion through the vessel lumen. In addition, patients with RA are at increased risk for thrombosis, further augmenting the risk. More studies investigating the clinical outcomes of stenting versus balloon angioplasty without stenting in patients with hypercoagulable risk factors need to be conducted. 
